# Ethical Principles for Physician Rating Sites

**DOI:** 10.2196/jmir.1899

**Published:** 2011-12-06

**Authors:** Daniel Strech

**Affiliations:** ^1^Institute for History, Ethics and Philosophy of MedicineCELLS - Centre for Ethics and Law in the Life SciencesHannover Medical SchoolHannoverGermany

**Keywords:** Physician rating sites, ethics, patient empowerment, patient-physician relationship

## Abstract

During the last 5 years, an ethical debate has emerged, often in public media, about the potential positive and negative effects of physician rating sites and whether physician rating sites created by insurance companies or government agencies are ethical in their current states. Due to the lack of direct evidence of physician rating sites’ effects on physicians’ performance, patient outcomes, or the public’s trust in health care, most contributions refer to normative arguments, hypothetical effects, or indirect evidence. This paper aims, first, to structure the ethical debate about the basic concept of physician rating sites: allowing patients to rate, comment, and discuss physicians’ performance, online and visible to everyone. Thus, it provides a more thorough and transparent starting point for further discussion and decision making on physician rating sites: what should physicians and health policy decision makers take into account when discussing the basic concept of physician rating sites and its possible implications on the physician–patient relationship? Second, it discusses where and how the preexisting evidence from the partly related field of public reporting of physician performance can serve as an indicator for specific needs of evaluative research in the field of physician rating sites. This paper defines the ethical principles of patient welfare, patient autonomy, physician welfare, and social justice in the context of physician rating sites. It also outlines basic conditions for a fair decision-making process concerning the implementation and regulation of physician rating sites, namely, transparency, justification, participation, minimization of conflicts of interest, and openness for revision. Besides other issues described in this paper, one trade-off presents a special challenge and will play an important role when deciding about more- or less-restrictive physician rating sites regulations: the potential psychological and financial harms for physicians that can result from physician rating sites need to be contained without limiting the potential benefits for patients with respect to health, health literacy, and equity.

## Introduction

Physician rating sites allow patients to evaluate their experience and satisfaction with their health care providers, similar to other service-oriented businesses. The ratings are posted online and are intended as a source of information for people searching for a physician.

In addition to the more than 30 private physician rating sites [1,2], more and more publicly hosted physician rating sites have gone online in the last 5 years. In 2007, the National Health Service (NHS) in the United Kingdom launched the NHS Choices website, which allows patients to evaluate both physicians and hospitals. In 2010, the largest German health insurer (AOK) launched its own portal, Arzt-Navi, which initially went through a test phase in 3 out of 16 German states and has been open to all German residents since May 2011. In the United States, the Hospital Compare site, maintained by the Centers for Medicare & Medicaid Services (CMS) and other publicly funded sites, provides information on the quality of care, but it does not yet permit patients to rate physicians [3].

Increasingly, research results are being published on fundamental characteristics of physician rating sites in their present condition in regard to their frequency, content, and user assessment patterns [1,2,4-7]. However, direct evidence of potential benefits and harms of physician rating sites is still lacking.

Only a handful of discussion papers on this topic have been published in scientific journals [3,8-10]. However, the media are increasingly discussing opportunities and challenges of physician rating sites. On the one hand, government and insurance company representatives often express their support of such concepts [11]. Ben Bradshaw, the former British Minister for Health, for example, criticized the general lack of transparency as an argument supporting physician rating sites, stating that “I would never think of going on holiday without cross-referencing at least two guide books and using Trip Advisor. We need to do something similar for the modern generation in healthcare.” Other critics have referred to evidence related to questions similar to those of physician rating sites [8]. They highlight that key clinical measures and outcomes are closely linked to patient satisfaction [12,13] and that systematic feedback changes doctors’ clinical performance [14]. Nevertheless, physician representatives tend to argue against physician rating sites. Laurence Buckman, Chairman of the British Medical Association’s General Practitioners Committee, fears that physician rating sites could compromise physicians: “A website on which people can slander or praise irresponsibly is the wrong approach” [11]. Likewise, Frank Ulrich Montgomery, President of the German Medical Association, described these websites as “platforms for denunciation” [15].

Taking the current state of discussion and scientific analysis of physician rating sites into account, health policy decision making, with respect to the implementation and regulation of physician rating sites, is challenging for at least two major reasons: (1) the lack of outcomes research in the field of physician rating sites, and (2) the controversial but poorly structured (ethical) debate on the pros and cons of physician rating sites.

This paper has two aims. First, it aims to structure the ethical debate around the basic concept of physician rating sites—that is, allowing patients to rate, comment, and discuss physicians’ performance, online and visible to everyone. This provides a more thorough and transparent (and therefore more reasonable) starting point for further discussion and further decision making on physician rating sites: what should physicians and health policy decision makers take into account when discussing the basic idea of physician rating sites and its possible implications for the physician–patient relationship? Second, it discusses where and how the preexisting evidence from the partly related field of public reporting of physician performance can serve (at least) as an indicator for specific needs of evaluative research in the field of physician rating sites.

While this paper focuses on the preceding ethical discussion concerning the basic concept of physician rating sites, it does not analyze the wide range of issues surrounding the safety and validity of information provided by physician rating sites. It also does not analyze the various judicial aspects of physician rating sites such as ownership or liability, for example. Needless to say, controversy in eHealth ethics often relates to these implementation issues [16] and they are also relevant for physician rating sites. The issues related to the implementation and management of physician rating sites, however, become relevant and need to be discussed thoroughly after the basic concept of physician rating sites has been generally accepted by physicians and health policy decision makers (as is the case with the basic concept of online patient information about diseases and treatment options, for example).

The following analysis is based on three ethical principles that are presented in widely acknowledged ethical frameworks for health care and health policy (eg, [17,18]). The two major reasons for choosing these rather general ethical principles are the following: (1) the discussion on physician rating sites is still in its beginnings, and a too-specific framework (eg, specific eHealth ethics frameworks [19,20]) runs the risk of excluding relevant ethical issues and arguments, and (2) because the structure provided here aims to inform health care professionals and health policy decision makers that are not always trained in ethics, it is helpful to frame the analysis with well-known ethical principles.

The three substantial ethical principles are (1) patient (and physician) welfare, (2) patient autonomy, and (3) social justice. The analysis of these substantial ethical principles is complemented by a brief description of procedural criteria that aims to improve the fairness of the health policy decision making concerning physician rating sites.

## Physician Rating Sites and Patient Welfare

Assuming that there are outcome-related quality differences between physicians and that physician rating sites can detect these differences to a certain degree, then physician rating sites could aim to improve health outcomes (patient welfare). One way to realize this goal is to make doctors aware of aspects of their work in need of improvement as identified by physician rating sites so that adjustments can be implemented in practice [14]. Second, patients who obtain information from physician rating sites are probably more likely to choose better-quality physicians and reap health benefits as a result. However, direct evidence that supports or negates these assumptions is lacking.

Can evidence from other fields be reasonably transferred to physician rating sites? The evidence related to the effects of public reporting of physician performance, based on either specific quality indicators or public report, is mixed [21]. Few researchers have examined the effects of public reporting on the actual delivery of health care [22,23] and even fewer have investigated how report cards might improve care [24,25]. Paradoxically, there is some evidence that public report cards can actually reduce quality by leading physicians to select patients based on risk profiles that best suit the specific quality indicator, which is critical for the interpretation of physician rating sites [26,27]. It is questionable, however, whether this scenario can be transferred to physician rating sites. Nevertheless, opponents of physician rating sites suggest that patients could be led to award more positive ratings based on nonmedical factors, such as pleasant waiting room music, or even persuasion by the physician.

Although physician rating sites cannot measure positive health outcomes directly, the presence of physician rating sites and the resulting transparency of medical performance could result in a (measurable) improvement in public trust in the system [21,28]. However, evidence either in support of or against this assumption is also lacking.

## Physician Rating Sites and Patient Autonomy

Besides the direct improvement in health outcomes, another intervention goal of physician rating sites that is closely linked to the ethical principle *respect of patient autonomy* can be grouped under the heading *improvement of health literacy* [29,30]. According to the World Health Organization’s definition, health literacy is “the cognitive and social skills which determine the motivation and ability of individuals to gain access to, understand, and use information in ways which promote and maintain good health” [31]. Improving health literacy empowers and thereby respects patient autonomy in making various health-related choices. Physician rating sites could potentially influence health literacy on three different levels [29] (see Table 1 [29,32]).

**Table 1:** The potential impact of physician rating sites on the three levels of health literacy

Level	Potential impact
Functional	People are able to process simple information that can help them find a specialist physician who understands medical procedures. Obviously, physician rating sites could promote this functional level of health literacy by providing a wide range of information; conversely, they could counteract it by disseminating false or at least biased information (for example, by putting only those physicians at the top if the list who bought a premium account offered by the specific physician rating sites).
Interactive	Through the development of enhanced cognitive and social skills and structures, this level of health literacy allows patients to play a more active role in interactions with their health environments. Physician rating sites could improve this interactive level—for example, if physician rating sites were to serve as a navigational tool with which patients are better able to steer through the health care system and enhance their communication and exchange of knowledge about specific physicians (or hospitals) from peer to peer (for example, by offering open text options at physician rating sites that allow users to describe in a more narrative style how they experienced the performance of a certain physician).
Critical	People have the ability to question so-called standards and to critically evaluate health-related information [29]. It would be practically relevant if, in general, the exchange of information between patients (peer-to-peer) regarding specific physicians and health care facilities would lead to the development of an increasingly higher and more discriminating level of quality assessment of health care through patients themselves. For example, physician ratings could cover aspects of health care quality that other patients have not sought before, thus providing the possibility to expand patients’ horizons in terms of quality assessment. Furthermore, users of physician rating sites could post ratings of which physician reviews were more or less helpful to them or may have even been false and misleading. See, for example, the concept of labeling—that is, to provide information about information (meta-information), which can be either descriptive or evaluative [32].

## Physician Rating Sites and Physician Welfare

Alongside the consideration of potential benefits and harms of physician rating sites for patients, the process of ethical decision making should also address the possible side effects for physicians. In particular, it should take into account the possible emotional and psychological burdens for physicians, as well as potential financial damages to those physicians who are not rated as well. In addition to the concern of defamation of individual physicians, another broader concern arises that physicians are discussed and furthermore rated not only based on their professional skills but also as a person. Refer to the assessment from the President of the German Medical Association, Frank U Montgomery, that “The only people who speak up on the Internet are those with an extreme opinion” (translation by the author) [15]. Buckman (see above) pointed his arguments in the same direction. Whether the fears of physician representatives are justified is more than questionable. Recent evaluations of rating tendencies from the United States and Germany demonstrate that the majority of reviews in physician rating sites appear to be positive [1,33].

Nevertheless, the potential harm to physicians should be taken seriously. For example, making the ratings first available when they have reached a certain baseline number (eg, 5–10) reduces the impact of extreme opinions, and peer review allows for the differentiation and elimination of defamations. However, when the baseline number or the peer-review requirements are set too high, then potential benefits for patients could be hindered. An ethical and regulative challenge is depicting the differentiation between disproportionate defamation on the one hand and relevant critique on the other hand. The criteria that physician rating sites use for these differentiation tasks (including examples of ratings classified as *defamation* or *relevant critique*) should be made transparent to the users. Furthermore, eliminating overly negative ratings without eliminating overly positive ratings will create a general bias toward more positive ratings and will decrease the differentiation between physicians. See also the section below on legitimacy of decision making in the implementation and regulation of physician rating sites.

## Physician Rating Sites and Social Justice

If we accept the assumption, as discussed above, that physician rating sites could have a positive effect on patient welfare as well as on the advancement of health literacy, then they could also have an impact on equal opportunity among the different socioeconomic groups within one society [30,34]. For reasons of equity, one should investigate whether all socioeconomic groups that could benefit from physician rating sites have unrestricted access to the Internet. The Internet as a source of information regarding the quality of physicians is relatively accessible in comparison with alternative forms of media (print media and personal contact). The relativity arises as a result of the contrast between possible effective alternatives. Arguably, one of the most effective available options to find a good physician is to ask friends or relatives in the medical profession to recommend a colleague. Thus, it is indisputable that physicians as a social group have structural advantages in the search for a new physician due to insider information received from colleagues.

Even though the Internet is widely accessible, one must consider remaining financial and nonfinancial access barriers, such as income, culture, gender, and age. Effective use of physician rating sites remains primarily dependent on the cognitive and intellectual capabilities of the users. This phenomenon could negatively affect the already-existing health inequalities between more- and less-educated groups (inequity).

A further issue to be considered are effects that have been observed in the context of public reporting of quality indicators [21]. If quality indicators such as satisfaction with care are correlated with race and socioeconomic status [35,36], then physicians may shy away from treating some groups of patients out of fear of being penalized by relatively poor ratings in physician rating sites.

## Legitimacy of Decision Making in the Implementation and Regulation of Physician Rating Sites

Decisions regarding the implementation or regulation of physician rating sites through public institutions (eg, NHS Choices in the United Kingdom or statutory health insurers in Germany) are associated with inevitably complex issues. Such issues cannot be solved by reference to an ultimate ethical principle [16]. Whenever health care institutions are confronted with the challenges of ethical assessments, they increase the legitimacy of their decisions when certain minimal requirements for a fair decision-making process are met [34] (see Table 2 [37-39]).

**Table 2:** Basic conditions for a fair decision-making process concerning the implementation and regulation of physician rating sites

Condition	Implication
Transparency	Empirical information and normative arguments that were relevant for the decision-making process on more- or less-restrictive regulation of physician rating sites should be made available to the public.
Justification	Decisions should be based on a relevant rationale. Relevant reasons are especially those that explicitly and comprehensibly ascribe to the above-described ethical criteria: patient and physician welfare, autonomy, and justice.
Participation	Subjective evaluations that are part of the decision-making process are inevitable due to the complexity of the question. The legitimacy of such subjective evaluations increases when the affected populations (here patients, physicians, and insurance agents) have been given the opportunity to participate and to provide relevant empirical information and normative arguments [37,38].
Minimizing conflicts of interest	Decisions on the implementation or regulation of physician rating sites should be regulated in order to avoid as many conflicts of interest as possible [39]. Conflicts of interest exist, for example, if the decision maker him- or herself benefits from any financial advantages on decisions made for or against any particular forms of regulation of physician rating sites.
Openness for revision	Every decision should be open for revision provided that better normative arguments or better evidence on the effects of physician rating sites is available.

## Discussion

The previous sections specified fundamental ethical principles relevant to the discussion of the basic concept of physician rating sites (allowing patients to rate, comment on, and discuss physicians’ performance—online and visible to everyone). The specified ethical principles should be recognized when the various stakeholders in the field of physician rating sites (physicians, patients, politicians, insurance companies, owners of private physician rating sites, and others) develop their viewpoints toward the basic concept of physician rating sites. These principles should also play a crucial role when decisions on the implementation and (more- or less-restrictive) regulation of physician rating sites are made. Even when thorough empirical evaluations of potential unknown effects of physician rating sites are strongly required, drawing on plausibility and normative arguments is unavoidable for justifying (regulatory) decisions regarding physician rating sites. The aforementioned basic conditions for a fair decision-making process are particularly relevant under such conditions of normative complexity and insufficient evidence (uncertainty).

In the opinion of the author, two issues present a special challenge and should play an important role when deciding about more- or less-restrictive physician rating sites regulations. First, the potential psychological and financial harms to physicians need to be contained without limiting the potential health and health literacy benefits for patients. In many countries the medical profession enjoys privileges such as strong advocacy groups and special social facilities. Thus, the denial of transparency on patient experiences and satisfaction (with physician performance) requires a strong rationale. Second, in light of the unequal distribution of health opportunities, particularly due to discrepancies in health literacy, possible countermeasures (such as physician rating sites) are highly relevant. Measures to improve public physician rating sites (such as NHS Choices and the AOK website) should be specifically tailored to the needs of vulnerable subgroups of the population. Preferably, aspects such as accessibility and the clarity of information should be evaluated and further improved.

If more general health policy discussions on the acceptance or rejection of the basic ideas of physician rating sites have been settled, further analyses need to focus on the ethical aspects of adequate implementation and management of such websites. Issues such as data privacy and validity, as well as ethical guidelines such as the e-Health Code of Ethics, will then play an important role [19,20,32].

## Abbreviations

NHS: National Health Service
